# Cross-Sectional Analysis of Human Papillomavirus Infection and Cytological Abnormalities in Brazilian Women

**DOI:** 10.3390/pathogens12010148

**Published:** 2023-01-16

**Authors:** Luis Fernando Lasaro Mangieri, Fernando Cezar-dos-Santos, Kleber Paiva Trugilo, Maria Angelica Ehara Watanabe, Rafaela Roberta de Jaime Curti, Eliza Pizarro Castilha, Sarah Lott Moretto, Caroline Yukari Motoori Fernandes, Janaina Nicolau de Oliveira, Karen Brajão de Oliveira

**Affiliations:** 1 Department of Gynecology and Obstetrics, Health Sciences Center, State University of Londrina, Londrina 86057-970, PR, Brazil; 2 Department of Pathological Sciences, Biological Sciences Center, State University of Londrina, Londrina 86057-970, PR, Brazil

**Keywords:** HPV, cervical cancer, risk factors, squamous intraepithelial lesions, cross-sectional study, cytological abnormalities

## Abstract

The aim of this study was to determine the incidence of infections and cytological abnormalities and to investigate possible predisposing factors such as sociodemographic characteristics, sexual behavioral habits, and gynecological and obstetric backgrounds. Between 2013 and December 2016, a cross-sectional study was conducted among 429 consenting women, from whom cervical samples were tested for the presence of Human papillomavirus (HPV) by polymerase chain reaction (PCR). Susceptibility to HPV infection was assessed by binary logistic regression in light of possible predisposing factors, which were collected using a questionnaire. In our sample population, the prevalence of HPV infection was 49%; high-risk types had a higher prevalence of 89.1%. A larger proportion of HPV-infected women were under 25 years of age, were single, and had monthly incomes up to minimum wage. Multivariate binary logistic regression analysis showed that age younger than 25 years increased the odds of infection fivefold, while a monthly income of one to three minimum wages provided protection against HPV infection, even if the women were married or had a cohabiting partner. In the HPV-positive group, squamous intraepithelial lesions (SIL) occurred more frequently in women who earned up to one minimum wage monthly, but a monthly income of one to three minimum wages protected against the development of SIL. The results suggest that age, marital status, and monthly income are important cofactors for HPV infection and the development of SIL.

## 1. Introduction

Cervical cancer (CC) is the third most common cancer in women worldwide, with an estimated 570,000 new cases in 2018, of which more than 85% occurred in less developed regions [1]. In Brazil, cervical cancer is also the third most common cancer in women, with more than 16,500 new cases annually [2]. Human papillomavirus (HPV) infection plays a central role in the development of cervical cancer and can reach a prevalence of 99.7% in cervical cancer samples [3]. It has been clearly demonstrated that high-risk HPV infection is necessary to promote progressive cell transformation leading to squamous intraepithelial lesions (SILs) and cervical cancer [4]. Although many women develop HPV infections of the cervix, studies support the interpretation that most HPV infections are only transiently detectable and do not lead to dysplasia or CC [5]. The natural history of HPV infection is not clear, but several cofactors are thought to promote the development and progression of SIL and CC. Viral factors such as HPV genotype, viral load, and coinfection with many HPV types are associated with abnormal cytologies in women [6]. In addition, sexual behavior and environmental factors, including hormonal contraceptives, tobacco smoking, parity, and coinfection with other sexually transmitted pathogens, especially *Chlamydia trachomatis* (CT), are associated with disease progression [2,7].

The HPV prevalence in cervical specimens in Brazil determined in a systematic review and meta-analysis study was 25.41% with a prediction interval of 7.17% to 60.04% depending on the population and geographical area studied [8]. Epidemiological data on the prevalence of HPV in the state of Paraná are still scarce. A large study was conducted in the city of Paiçandu, in northwestern Paraná, and the overall prevalence of HPV deoxyribonucleic acid (DNA) found in this area was lower than the levels found in studies conducted in other Brazilian regions that also used polymerase chain reaction (PCR) [9]. A recent study conducted in Maringá, another city in northwestern Paraná, found an HPV prevalence of 33.8% [10]. However, it is necessary to extend this study to other cities in the region, since there is a great need for medical care in the North Paraná region.

The aim of this cross-sectional study was to determine the incidence of HPV infection and cytological abnormalities and to investigate possible predisposing factors such as sociodemographic characteristics, sexual behavioral habits, and gynecological and obstetric history.

## 2. Materials and Methods

### 2.1. Study Design and Sample Collection 

The women participating in the study were from the city of Londrina and surrounding small towns. This large city is located in the northern region of the Brazilian state of Paraná (southern region of the country) and is more than 300 km from the respective capital (Curitiba).

Cervical cell samples were randomly collected between 2013 and December 2016 from 429 women who presented for outpatient appointments at the colposcopy outpatient clinic of the Intermunicipal Health Consortium of Middle Paranapanema and the College Hospital and Clinical Center of the College of Londrina, and who had undergone cervical cancer screening cytology at two primary healthcare facilities (Municipal Health Centers Dr. Justiniano Clímaco da Silva and Dr. Paulo Roberto Moita da Silva). The women included in the present study were participating in cervical cancer prevention programs, since, according to the Brazilian guidelines for cervical cancer screening, routine cytological examination is recommended for women aged 25 to 64 years who are sexually active [11]. After sample collection for cytology, cytobrushes were stored in 2 mL of ethylenediaminetetraacetic acid (EDTA)–Tris (TE) buffer (10 mM Tris hydrochloride (HCl), 1 mM EDTA) pH 8.0) at −20 °C until analysis. Peripheral blood was collected in sterile syringes containing EDTA and stored at −20 °C until analysis. Patients were interviewed using a structured questionnaire to collect sociodemographic data, sexual behavior data, and gynecologic and obstetric data. The women who participated in this study had not been vaccinated at the time the samples were collected. The HPV vaccine has only been distributed by the Unified Brazilian Health System since March 2014. Distribution of the vaccine was limited to girls aged 11–14 years [12].

### 2.2. Cervical Cytology

Cervical cancer smears obtained during screening in healthcare units were sent to the public health laboratory. They were evaluated and reported according to the diagnostic criteria of the Bethesda system (2014). SIL was defined as low-grade squamous intraepithelial lesions (LSIL), high-grade squamous intraepithelial lesions (HSILs), atypical squamous cells of undetermined significance (ASC-US), HSILs that could not be excluded (ASC-H), or cervical carcinoma (CC), while controls were negative for intraepithelial lesions or malignancy and all SIL types were excluded [13].

### 2.3. DNA Extraction

Genomic DNA from cervical cells was extracted from Cytobrush samples using DNAzol (Invitrogen Inc., Carlsbad, CA, USA) according to the manufacturer’s instructions. DNA concentrations were measured at 260 nm using the NanoDrop 2000c™ spectrophotometer (Thermo Fisher Scientific, Waltham, MA, USA), and purity was assessed by the 260 nm/280 nm ratio. DNA samples were then stored at −20 °C.

### 2.4. HPV Detection by Polymerase Chain Reaction (PCR)

HPV was detected by PCR using primers MY09 (5′–CGTCCMAARGGAWACTGATC–3′) and MY11 (5′–GCMCAGGGWCATAAYAATGG–3′), which amplify a conserved region of approximately 450 base pairs (bp) of the L1 HPV gene [14]. Reaction conditions were 190 nM dNTPs, 500 nM of each primer, 2 mM MgCl2, 1× buffer, approximately 80 ng of DNA, and 1.25 U of Taq polymerase (InvitrogenTM, Carlsbad, CA, USA), with an annealing temperature of 55 °C. This method was chosen because it targets very small fragments and is, therefore, more sensitive than several other molecular techniques. Co-amplification of the human b-globin gene (268 bp) was performed as an internal control using primers GH20 (5′–GAAGAGCCAAGGACAGGTAC–3′) and PC04 (5′–CAACTTCATCCACGTTCACC–3′) under the same conditions as HPV PCR. A negative control (no DNA) was also added to all reactions to ensure that no contamination was present, as well as a positive control consisting of a cervical adenocarcinoma cell line containing an integrated HPV18 genome (HeLa). The PCR product was analyzed by electrophoresis in a 10% polyacrylamide gel stained with silver nitrate.

### 2.5. PCR Detection of Chlamydia Trachomatis (CT)

PCR assay for detection of CT was performed using primers specific for the gyrA gene: forward primer C2 (5′–TGATGCTAGGGACGGATTAAAACC–3′) and reverse primer C5 (5′–TTCCCCTAAATTATGCGGTGGAA–3′), as described previously [15]. Co-amplification of the human b-globin gene was also performed as an internal control. For each set of tests, a DNA pool extracted from cervical cells infected with CT was used as a positive control, and “no DNA” was used as a negative amplification control. The 463 bp amplicons were analyzed by electrophoresis in a 10% polyacrylamide gel stained with silver nitrate.

### 2.6. Amplicons Sequencing

To confirm primer specificity, some amplicons of both HPV and CT DNA were purified using the PureLinkTM PCR Purification Kit (Invitrogen) according to the manufacturer’s instructions. The sequencing reaction was performed using the BigDye^®^ Terminator v3.1 Cycle Sequencing Kit (Applied Biosystems^®^, Foster City, CA, USA), 50 ng of DNA template, and 5 µM primer (forward or reverse) in a final volume of 10 µL. PCR conditions were as follows: 10 s at 95 °C, 30 cycles of 20 s at 95 °C, 20 s at 50 °C, and 60 s at 60 °C. Amplicons were sequenced in a 24-capillary 3500xl Genetic Analyzer (Applied Biosystems, Life Technologies, Thermo Fischer Scientifc, Foster City, CA, USA ^®^). Percentage identity was determined using the program BLAST by comparing the DNA sequences of the amplicons to known HPV L1 or gyrA nucleotide sequences in the GenBank databases.

### 2.7. Statistical Analysis

Associations between categorical variables were analyzed with the chi-square test (χ^2^) or Fisher’s exact probability test, where appropriate, and expressed in absolute numbers (n) and percentages (%). Differences between categories of the same variables were assessed using the Mann–Whitney test and expressed as the median and interquartile range (IQR) 25–75%. The odds ratio (OR) and 95% confidence interval (95% CI) were determined. Binary logistic regression analysis was used to determine the significant predictors of HPV infection compared with controls (uninfected), and multinomial logistic regression was used to analyze the risk factors for the presence of SIL compared with controls (normal cervical cytology) in HPV-infected women. In both analyses, sociodemographic and sexual behavioral, gynecologic, and obstetric factors associated with HPV infection or a SIL diagnosis in the bivariate analysis were each included as explanatory variables. All tests were two-sided, and a significance level of α = 0.05 was assumed. Analyses were performed using IBM SPSS Statistics 22.0 software (SPSS Inc. 2013. Chicago, IL, USA).

## 3. Results

Some samples were sequenced to confirm primer specificity. The identity percentage was determined using the program BLAST, and the sequences obtained for HPV and CT demonstrated 100% identity with the L1 sequence, AJ617545.1, and the GyrA CT subunit, JN795357.1, respectively.

The sociodemographic characteristics of the HPV-negative and -positive women are shown in Table 1. There were no significant differences between these groups in self-reported ethnicity (*p* = 0.18), schooling (*p* = 0.83), knowledge of HPV (*p* = 0.50), and viral transmission (*p* = 0.63). However, HPV-infected women had a lower mean age, 33 (25–46) years, than uninfected women, 43 (32–52) years (*p* < 0.001). To determine in which age range HPV infection was more common, the continuous variable “age” was divided into “age ranges”, as shown in Table 1. Thus, compared with the control group, a greater proportion of HPV-infected women were younger than 25 years (*p* < 0.001), were single (*p* < 0.001), had a reported monthly income of up to minimum wage (*p* = 0.018), and were smokers (*p* = 0.014).

Table 2 shows the analysis of HPV-negative and -positive groups according to sexual behavior and gynecologic and obstetric characteristics. Age at menarche (*p* = 0.21), number of sexual partners in the past 6 months (*p* = 0.051), oral contraceptive use (*p* = 0.13), condom use (*p* = 0.44), spontaneous abortion (*p* = 0.27), and CT infection (*p* = 0.056) were not statistically different between the HPV-positive group and the control group. However, HPV infection was associated with first sexual intercourse before age 18 (*p* = 0.012), at least four lifetime sexual partners (*p* < 0.001), and pregnancy that did not occur (*p* = 0.008).

The occurrence of CT infection was more common in women who tested positive for HPV infection. Although this difference was not statistically significant, the *p* = 0.056 value may indicate a tendency toward a higher CT prevalence in HPV-infected women.

Considering the number of existing cases of the analyzed conditioning variables in relation to the total population sample analyzed in this study, the characterization of the sample in terms of prevalence of HPV infection was 49%, with a higher prevalence of these high-risk types (89.1%) compared with low-risk types (8.0%) and undetermined risk (2.9%). The prevalence of infection with *C. trachomatis* was 4.7%, and the proportion of coinfections with HPV and *C. trachomatis* in cervical lesions was 7.2% and in healthy individuals was 2.2% in the population sample studied. The presence of cervical lesions was 25.2%. When cervical lesions were stratified according to the degree of involvement, a prevalence of 7.3% for LSIL, 17.7% for HSIL, and 1.2% for cancer could be observed.

To test whether these significant variables (age, marital status, monthly income, smoking status, age at first sexual intercourse, number of lifetime sexual partners, and number of pregnancies) were independently associated with HPV infection, a binary logistic regression analysis was performed with a control group as the reference category. A direct association was found for age less than 25 years, which increased the odds of acquiring the virus approximately fivefold (OR = 4.92; 95% CI = 1.67–14.52; *p* = 0.004), whereas both married and lifetime partners (OR = 0.45; 95% CI = 0.23–0.88; *p* = 0.020) and a monthly income of one to three minimum wages (OR = 0.59; 95% CI = 0.36–0.95; *p* = 0.030) provided protection against HPV infection (Table 3).

After verifying which variables were directly and independently associated with HPV infection, the HPV-positive group was categorized into normal cytology, LSIL presence, and HSIL presence according to cytological results to analyze the influence of sociodemographic and sexual behaviors, as well as gynecological and obstetric characteristics, on the development of SIL. Of all independent variables studied, the presence of HSIL was associated with age <25 years (*p* = 0.002), oral contraceptive use (*p* = 0.010), and spontaneous abortion (*p* = 0.010) (data not shown). The presence of LSIL was associated with <25 years (*p* = 0.002), a monthly income of up to minimum wage (*p* < 0.001), and more than four lifetime sexual partners (*p* = 0.019). In multinomial logistic regression analysis with normal cytology as the reference category, all age categories >25 years and spontaneous abortion (OR = 4.84; 95% CI = 1.72–13.60; *p* = 0.003) were independently associated with the risk of developing LSIL (Table 4). In the analysis of HSIL, only >4 lifetime sexual partners (OR = 3.41; 95% CI = 1.35–8.61; *p* = 0.009) was an independent risk factor (Table 4).

## 4. Discussion

In this cross-sectional study, the incidence of HPV infection by PCR and predisposing factors for infection and cytological abnormalities were determined in women who received care in the public health system (SUS) in Londrina, Paraná State, Brazil. To our knowledge, this is the first epidemiological and molecular report to investigate HPV prevalence in this region.

This study found a prevalence of 49.0% HPV infection in the women studied, of which 89.1% were high-risk types, 8.0% were low-risk types, and 2.9% were of undetermined risk (0.9%). However, we were unable to perform it in all our sampling, configuring a limitation of our study. HPV infection was associated with four sociodemographic characteristics (age, marital status, smoking status, and monthly income), two sexual behavior variables (age at first sexual intercourse and lifetime sexual partner), and one gynecologic and obstetric aspect (parity).

Only those younger than 25 years, marital status with a married or civil partner, and monthly income of one to three minimum wages (Table 1) were independently associated with HPV infection in a multivariate model.

The association between young age and HPV infection is well established in the literature as an independent factor, showing that HPV prevalence is age-specific worldwide for both low-risk types (LR-HPV) and high-risk types (HR-HPV) [16]. Teenagers and young women are more sexually active and more susceptible to pathogen infections because they have a particular cervical anatomy that manifests cervical ectopy in addition to early maturation stages, making them vulnerable to both trauma and infection, especially in the developing transformation zone [17].

Single women were significantly more likely to have HPV in the HPV group (Table 1), which is consistent with the findings of Foliaki et al. [18]. In addition, a significantly higher proportion of married women was observed in the control group than in the HPV-infected group (Table 1), presumably because they have a steady partner [19].

It is also known that multiparity, which becomes more likely in women who start having sex earlier, is associated with a higher risk of exposure of women to HPV infection and other cofactors [7,20].

Tobacco smoking was also associated with HPV infection in this study (Table 1). Several compounds from cigarette smoke such as nicotine (and its major metabolite cotinine) and carcinogenic tobacco-specific N-nitrosamines were detected in cervical mucus, highlighting the synergistic effect between cigarette smoking and HPV infection [21]. Another tobacco-related carcinogen, benzo[a]pyrene (BaP), can interact with HPV, modulating the life cycle of the virus and promoting its synthesis [22]. Tobacco smoking may also reduce the density of Langerhans cells, affecting local immune surveillance in the cervix [23].

Women who reported a monthly income at or below the minimum wage were more likely to be infected with HPV (Table 1). These results make sense considering that a monthly income of less than a Brazilian minimum wage (i.e., low socioeconomic status) is strongly associated with HPV infection in studies in the Brazilian northeast and abroad [24]. In fact, sociodemographic data show the social inequalities associated with the high risk of HPV infection leading to cervical cancer, as the virus is more prevalent in public health facilities than in private clinics [25]. In this context, the lack of knowledge about HPV, as well as its prevention and transmission, is a factor that should be considered in women with less education [26].

We found an independent association between monthly income of one to three minimum wages and protection from SIL among women infected with HPV (Table 3). These results suggest that a higher monthly income of women denotes that they are less susceptible to developing cervical lesions. This information confirms the observation that (poor) economically disadvantaged women and girls in many parts of the world are more vulnerable to sexually transmitted diseases due to limited access to economic and educational resources and to prevention information and tools [27].

Regarding sexual behavior and gynecologic and obstetric aspects, an association was found between HPV infection and first sexual intercourse before the age of 18 years and at least four sexual partners during life (Table 2). As mentioned earlier, a physiologically ectopic and immature genital tract may explain the predisposition to HPV infection in young women. In addition, a high number of sexual partners is an important risk factor for acquiring HPV [20]. We also observed an inverse association for one pregnancy with HPV infection (Table 2), but not for more than two pregnancies. High parity is consistently associated with susceptibility to HPV infection, and hormonal, traumatic, and immunologic mechanisms are thought to play a role in this association [28].

Our study showed a tendency for association between HPV infection and the intracellular bacterium *Chlamydia trachomatis* (CT) (Table 2). This tendency was also reported by Nonato et al. (2016) [29]. Limitations related to the small number of women with HPV/CT coinfection may have contributed to these results in both studies. In this context, the association between CT genital infections and HPV has been most thoroughly studied in the development of CC. CT Infection facilitates infection and reinfection with multiple HPV types, allows viral persistence, and increases the risk of developing CC in coinfection cases [30]. In fact, evidence suggests that CT and HPV share the same transmission route and risk factors [30].

Having more than four sexual partners over a lifetime was associated with the development of HSIL, and spontaneous abortion was associated with the development of LSIL, which is consistent with other meta-analysis of epidemiological studies demonstrating the involvement of these factors from HPV infection to tumor occurrence [31,32].

Cervical cancer screening is effectively performed in Brazil through the Pap smear, a method that has led to a decrease in CC incidence in the country over the last five decades. Nevertheless, new cases of CC are detected every year, and mortality from this cancer is still alarming worldwide [33]. It is not difficult to explain that this failure of screening is due to the subjective and poorly reproducible nature of cervical cytology. The Pap test has limited sensitivity, requires regular repetition to achieve the desired efficacy, and may suffer from interobserver variability [9].

Although this was a cross-sectional study that did not allow us to draw conclusions about causal relationships, we would like to use our cohort and several reports published in recent years to draw attention to the fact that diagnosis by HPV DNA using polymerase chain reaction in combination with cytologic analysis allows more accurate detection of HPV and lesions, even when abnormalities are not detected in the Pap smear. 

## 5. Conclusions

In this article, we described the HPV occurrence in the northern region of the Brazilian state of Paraná and important cofactors for HPV infection and the development of SIL in our population-based study. In this sense, we hope to contribute to a better characterization of HPV epidemiology and to the implementation of public health policies in Brazil.

## Figures and Tables

**Table 1:** Sociodemographic profile of HPV-positive and -negative women.

Variable ^a^	HPV-Negative		HPV-Positive		*p*
	n	(%)	n	(%)	
Age range (years)					<0.001 **
<25	12	(5.6)	46	(22.1)	
25–34	53	(24.5)	63	(30.3)	
35–44	54	(25.0)	44	(21.2)	
45–54	61	(28.2)	28	(13.5)	
>54	36	(16.7)	27	(13.0)	
Self-reported ethnicity					0.189
Caucasian	116	(54.5)	95	(48.0)	
Not Caucasian	97	(45.5)	103	(52.0)	
Schooling level ^b^					0.836
Until incomplete fundamental education	65	(30.5)	62	(31.3)	
Complete fundamental education	26	(12.2)	24	(12.1)	
Incomplete secondary education	29	(13.6)	29	(14.6)	
Complete secondary education	69	(32.4)	67	(33.8)	
Incomplete higher education	7	(3.3)	7	(3.5)	
Complete higher education	17	(8.0)	9	(4.5)	
Marital status					<0.001 **
Single	22	(10.1)	52	(24.8)	
Married/Civil partner	158	(72.5)	120	(57.1)	
Divorced	27	(12.4)	24	(11.4)	
Widowed	11	(5.0)	14	(6.7)	
Monthly income ^c^					0.018 *
≤1 minimum wage	56	(26.3)	75	(39.3)	
1–3 minimum wages	140	(65.7)	101	(52.9)	
>3 minimum wages	17	(8.0)	15	(7.9)	
Smoking status					0.014 *
No	181	(83.8)	151	(74.0)	
Yes	35	(16.2)	53	(26.0)	
Knowledge about HPV					0.509
No	42	(19.5)	48	(24.1)	
Have ever heard	117	(54.4)	100	(50.3)	
Yes	56	(26.0)	51	(25.6)	
Knowledge about transmission					0.631
No	97	(45.1)	94	(47.5)	
Yes	118	(54.9)	104	(52.5)	

Analysis by the two-sided chi-square (χ²) test with *p* < 0.05 set as significance level; data expressed as absolute number and percentage (%). * *p* < 0.01; ** *p* < 0.001. ^a^ For sociodemographic characteristics analysis between HPV noninfected and infected patients, not all 429 women were included, with variations depending on the characteristic analyzed. ^b^ Based on the Brazilian educational system. ^c^ Based on the Brazilian minimum wage (approximately 200.00 USD).

**Table 2:** Sexual behavioral and gynecological and obstetric characteristic profile of HPV-positive and -negative women.

Variable ^a^	HPV-Negative		HPV-Positive		*p*
	n	(%)	n	(%)	
Age at menarche (years)					0.213
≤12	98	(45.2)	106	(51.2)	
>12	119	(54.8)	101	(48.8)	
Age at first sexual intercourse (years)					0.012 *
≥18	110	(50.5)	79	(38.3)	
<18	108	(49.5)	127	(61.7)	
Sexual partners during the lifetime					<0.001 ***
1	87	(40.5)	45	(22.8)	
2–3	70	(32.6)	68	(34.5)	
4	58	(27.0)	84	(42.6)	
Sexual partners within the past 6 months					0.051
≤1	207	(99.0)	178	(95.7)	
>1	2	(1.0)	8	(4.3)	
Oral contraceptive					0.130
No	148	(69.2)	128	(62.1)	
Yes	66	(30.8)	78	(37.9)	
Condom					0.445
No	191	(88.8)	171	(86.4)	
Yes	24	(11.2)	27	(13.6)	
Number of pregnancies					0.004 **
0	19	(8.7)	37	(17.6)	
1	36	(16.4)	45	(21.4)	
>2	164	(74.9))	128	(61.0)	
Spontaneous Abortion					0.276
No	175	(79.9)	172	(81.9)	
Yes	44	(20.1)	38	(18.1)	
*C. trachomatis* infection					0.056
No	92	(94.8)	110	(87.3)	
Yes	5	(5.2)	16	(12.7)	

Analysis by two-sided chi-square (χ²) test or by Fisher’s exact test when appropriate with *p* < 0.05 set as significance level; data expressed as absolute number and percentage (%). * *p* < 0.05; ** *p* < 0.01; *** *p* < 0.001. ^a^ For sexual behavioral and gynecological and obstetric characteristics analysis between HPV noninfected and infected patients, not all 429 patients were included, with variations depending on the characteristic analyzed.

**Table 3:** Binary logistic regression between groups of HPV noninfected (reference) and infected women as dependent variables and explanatory variables.

Variable	*χ*²_Wald_	df	OR	CI_95%_	*p*
Age range (years)					
<25	8.32	1	4.92	(1.67–14.52)	0.004 **
25–34	3.22	1	2.10	(0.93–4.70)	0.073
35–44	8.34	1	1.26	(0.59–2.67)	0.549
45–54	15.26	1	0.69	(0.33–1.48)	0.346
>54	-	4	1.00	Reference	-
Marital status					
Single	-	3	1.00	Reference	-
Married/civil partner	5.42	1	0.45	(0.23–0.88)	0.020 *
Divorced	0.65	1	0.70	(0.29–1.67)	0.419
Widowed	0.01	1	1.07	(0.36–3.21)	0.904
Monthly income ^a^					
≤1 minimum wage	-	2	1.00	Reference	-
>1–3 minimum wages	4.70	1	0.59	(0.36–0.95)	0.030 *
>3 minimum wages	0.06	1	1.11	(0.47–2.67)	0.808
Smoking status					
No	-	2	1.00	Reference	-
Yes	0.91	1	1.31	(0.75–2.28)	0.341
Number of pregnancies					
0	-	4	1.00	Reference	-
1	8.93	1	0.40	(0.22–0.73)	0.003 **
>2	1.51	1	0.64	(0.31–1.30)	0.218
Age at first sexual intercourse (years)					
≥18	-	2	1.00	Reference	-
<18	0.001	1	0.99	(0.62–1.63)	0.980

df, degrees of freedom; OR, odds ratio; 95% CI, 95% confidence interval. * *p* < 0.05; ** *p* < 0.01; ^a^ Based on Brazilian minimum wage, approximately 200.00 USD.

**Table 4:** Binary logistic regression between HPV infected women with normal cytology (reference) and with SIL and explanatory variables.

**LSIL** **Variable**	***χ*²_Wald_**	**df**	**OR**	**CI_95%_**	***p***
Age range (years)					
<25	-	-	-	Reference	
25–34	4.09	1	0.22	(0.05–0.95)	0.043 *
35–44	3.95	1	0.195	(0.03–0.97)	0.047 *
45–54	5.05	1	0.11	(0.01–0.75)	0.025 *
>54	4.35	1	0.08	(0.00–0.08)	0.037 *
Marital status					
Single	-	-	-	Reference	-
Married/civil partner	0.32	1	0.87	(0.21–3.60)	0.858
Divorced	2.14	1	3.72	(0.64–21.63)	0.143
Widowed	2.99	1	6.07	(0.78–46.82)	0.084
Age at first sexual intercourse					
≥18	-	-	-	Reference	-
<18	1.10	1	0.55	(0.18–1.67)	0.293
Monthly income ^a^					
≤1 minimum wage	-	-	-	Reference	-
>1–3 minimum wages	0.79	1	0.46	(0.08–2.50)	0.372
>3 minimum wages	1.39	1	1.31	(0.04–2.12)	0.237
Sexual partners during the lifetime					
1	-	-	-	Reference	-
2–3	0.00	1	1.00	(0.28–3.48)	0.999
>4	0.16	1	1.76	(0.20–2.83)	0.688
Oral contraceptive					
No	-	-	-	Reference	-
Yes	0.92	1	1.67	(0.58–4.78)	0.336
Spontaneous Abortion					
No	-	-	-	Reference	-
Yes	8.95	1	4.84	(0.72–13.60)	0.003 *
**HSIL** **Variable**	**-*χ²*_Wald_**	**df**	**OR**	**CI_95%_**	***p***
Age range (years)					
<25	-	-	-	Reference	-
25–34	0.27	1	1.32	(0.46–3.71)	0.599
35–44	0.00	1	1.02	(0.33–3.14)	0.961
45–54	0.18	1	0.75	(0.21–2.66)	0.665
>54	0.11	1	0.77	(0.17–3.40)	0.737
Marital status					-
Single	-	-	-	Reference	-
Married/civil partner	0.42	1	1.08	(0.47–2.48)	0.838
Divorced	0.02	1	1.09	(0.36–3.29)	0.868
Widowed	0.35	1	1.60	(0.33–7.62)	0.551
Age at first sexual intercourse					
≥18	-	-	-	Reference	-
<18	0.00	1	1.00	(0.50–1.97)	0.994
Monthly income ^a^					
≤1 minimum wage	-	-	-	Reference	-
>1–3 minimum wages	0.13	1	1.34	(0.28–6.32)	0.711
>3 minimum wages	3.46	1	4.45	(0.92–21.52)	0.063
Sexual partners during the lifetime					
1	-	-	-	Reference	-
2–3	3.63	1	2.43	(0.97–6.06)	0.057
>4	6.73	1	3.41	(1.35–8.61)	0.009 **
Oral contraceptive					
No	-	-	-	Reference	-
Yes	0.89	1	1.40	(0.69–2.81)	0.343
Spontaneous Abortion					
No	-	-	-	Reference	-
Yes	0.20	1	0.81	(0.34–1.94)	0.650

df, degrees of freedom; OR, odds ratio; CI95%, 95% confidence interval. * *p* < 0.05; ** *p* < 0.01. ^a^ Based on Brazilian minimum wage, approximately 200.00 USD.

## Data Availability

Not applicable.
